# Dissecting Variation in Biomass Conversion Factors across China’s Forests: Implications for Biomass and Carbon Accounting

**DOI:** 10.1371/journal.pone.0094777

**Published:** 2014-04-11

**Authors:** Yunjian Luo, Xiaoquan Zhang, Xiaoke Wang, Yin Ren

**Affiliations:** 1 Key Laboratory of Urban Environment and Health, Institute of Urban Environment, Chinese Academy of Sciences, Xiamen, China; 2 The Nature Conservancy China Program, Beijing, China; 3 State Key Laboratory of Urban and Regional Ecology, Research Center for Eco-Environmental Sciences, Chinese Academy of Sciences, Beijing, China; Institute of Botany, Chinese Academy of Sciences, China

## Abstract

Biomass conversion factors (BCFs, defined as the ratios of tree components (i.e. stem, branch, foliage and root), as well as aboveground and whole biomass of trees to growing stock volume, Mg m^−3^) are considered as important parameters in large-scale forest biomass carbon estimation. To date, knowledge of possible sources of the variation in BCFs is still limited at large scales. Using our compiled forest biomass dataset of China, we presented forest type-specific values of BCFs, and examined the variation in BCFs in relation to forest type, stand development and environmental factors (climate and soil fertility). BCFs exhibited remarkable variation across forest types, and also were significantly related to stand development (especially growing stock volume). BCFs (except Stem BCF) had significant relationships with mean annual temperature (MAT) and mean annual precipitation (MAP) (*P*<0.001). Climatic data (MAT and MAP) collectively explained 10.0–25.0% of the variation in BCFs (except Stem BCFs). Moreover, stronger climatic effects were found on BCFs for functional components (i.e. branch, foliage and root) than BCFs for combined components (i.e. aboveground section and whole trees). A general trend for BCFs was observed to decrease and then increase from low to high soil fertility. When qualitative soil fertility and climatic data (MAT and MAP) were combined, they explained 14.1–29.7% of the variation in in BCFs (except Stem BCFs), adding only 4.1–4.9% than climatic data used. Therefore, to reduce the uncertainty induced by BCFs in forest carbon estimates, we should apply values of BCFs for a specified forest type, and also consider climatic and edaphic effects, especially climatic effect, in developing predictive models of BCFs (except Stem BCF).

## Introduction

Reducing uncertainties in estimates of forest biomass carbon storage and their changes is a prerequisite not only for resolving a long-standing controversy about the role of forests in the global carbon cycle [1], but also for improving the accuracy of forest carbon accounting [2], [3]. With a large number of statistically valid plots covering nearly all forest types with different site conditions, widely available forest inventory data (FID) are recognized as the best ground-based observations for identifying the magnitude and spatiotemporal patterns of forest biomass carbon storage and its changes at large scales (e.g. landscape, regional and global) [4], [5]. Moreover, FID-based estimates are usually used to calibrate ones obtained by remote sensing, modeling or their combinations [6], [7]. Unfortunately, traditional FID generally provides information on tree volume (e.g. merchantable volume or growing stock volume) but no available biomass data. Therefore, the requirement to accurately obtain FID-based biomass carbon storage and its changes draws attention in order to assess forest carbon stocks and their dynamics.

Generally, FID-based biomass carbon storages are estimated using a volume-to-biomass conversion method and carbon concentration (CC) [8], [9]. The method has gained prominence in biomass estimation [2], [8]. A convenient descriptor of the volume-to-biomass conversions is biomass conversion (or expansion) factors (BCFs) (i.e. the ratios of biomass to volume) [10]–[16]. BCFs are increasingly used in favor of volume-to-biomass conversion models (i.e. the biomass as a function of the volume) and the product of wood density times biomass expansion factors (i.e. ratios of some or all of tree biomass to stem biomass) [2]. BCFs have been identified as a large source of uncertainty in FID-based carbon estimates [8], because inventories of stem volumes tend to be precise [4], [17], and CC is a relatively constant variable (e.g. 0.45 and 0.50) [18].

Knowledge of sources of the variation in BCFs is crucial to reduce uncertainty in BCFs, thus FID-based carbon estimates. Furthermore, this has important implications for how resources should be invested to determine BCFs. Indeed many early studies used static values of BCFs [19]–[21]. Recent studies have resulted in significant advances in reducing the uncertainty of BCFs. They focused on tree age- and size-related variation in BCFs for specific forest types [11]–[16], [22], [23]. Now local-level evidences indicate that BCFs vary with site conditions (e.g. temperature, water and nutrients) [24]. However, the effects and relative importance of environmental factors on BCFs remains unclear at large scales (e.g. landscape and national).

China has diverse range of forest types, ranging from tropical rainforest to boreal forest [25]. It has a good representation of global biome heterogeneity and environmental gradients, and thus provides an excellent opportunity to explore possible sources of variation in BCFs. During recent years, a comprehensive forest biomass dataset of China has been compiled based on a literature survey. Our objective was to use the dataset to determine values of various BCFs specific to forest types, and then to explore the variation in BCFs in relation to forest type, stand development and environmental factors (climate and soil fertility). Our results will contribute to accurately determining the values and predictive models of BCFs, thereby improving the reliability of the estimates of forest biomass and carbon stocks.

## Materials and Methods

### The dataset and data categorization

To date, we have compiled a comprehensive forest biomass dataset of China from available published studies and previous datasets [26]–[28]. For details, see File S1. For calculating BCFs and exploring their possible sources of the variation, only paired data with both growing stock volume and biomass measurements (for at least tree components, i.e. stem, branch and foliage) were selected from our dataset. As a result, a total of 1,099 paired data from 252 sites were available for subsequent analyses. These sites showed a sound spatial distribution across China (Fig. S1 in File S2), ranging from −5.1 to 23.5°C in mean annual temperature (MAT) and from 223 to 2300 mm in mean annual precipitation (MAP).

According to dominant tree species and ecogeographical zones given by a Chinese Vegetation Classification System [25], we categorized these stands into 15 forest types with a sample size of at least 10 (Table 1). For details, see Table S1 in File S2. We also categorized these stands by leaf form into coniferous forest and broadleaved forest, by leaf lifespan into deciduous forest and evergreen forest, and by stand origin into natural forest and planted forest.

**Table 1:** Statistics of biomass conversion factors (BCFs, Mg m^−3^) for forest types (groups) in China *.

Forest type (group) †	*n*	Stem BCF	Branch BCF	Foliage BCF	Aboveground BCF	*n*	Root BCF	Whole BCF
All data	1099	0.505 (0.247)	0.137 (0.112)	0.096 (0.141)	0.740 (0.412)	796	0.172 (0.125)	0.890 (0.489)
Forest type (group)								
* Abies* and *Picea*	37	0.513 (0.226)	0.136 (0.110)	0.141 (0.277)	0.792 (0.573)	25	0.164 (0.108)	1.042 (0.775)
* Cunninghamia lanceolata*	239	0.394 (0.161)	0.086 (0.090)	0.136 (0.222)	0.617 (0.428)	194	0.152 (0.132)	0.741 (0.488)
* Cupressus* and *Fokienia*	22	0.451 (0.103)	0.111 (0.049)	0.111 (0.087)	0.674 (0.191)	18	0.141 (0.047)	0.799 (0.244)
* Larix*	85	0.512 (0.127)	0.119 (0.101)	0.044 (0.035)	0.674 (0.219)	38	0.186 (0.065)	0.915 (0.280)
* Pinus koraiensis*	35	0.427 (0.082)	0.140 (0.070)	0.102 (0.064)	0.668 (0.187)	33	0.156 (0.062)	0.830 (0.243)
* P. massoniana*	80	0.550 (0.177)	0.139 (0.125)	0.066 (0.078)	0.755 (0.321)	63	0.139 (0.067)	0.914 (0.391)
* P. tabuliformis*	105	0.442 (0.101)	0.165 (0.106)	0.121 (0.113)	0.733 (0.278)	74	0.149 (0.044)	0.861 (0.283)
Other temperate pines and conifers	47	0.567 (0.323)	0.230 (0.112)	0.131 (0.091)	0.933 (0.467)	39	0.225 (0.160)	1.110 (0.591)
Other subtropical pines and conifers	69	0.442 (0.123)	0.130 (0.077)	0.099 (0.084)	0.672 (0.228)	53	0.155 (0.090)	0.782 (0.260)
* Betula* and *Populus*	49	0.633 (0.410)	0.205 (0.168)	0.093 (0.126)	0.930 (0.645)	28	0.178 (0.155)	0.879 (0.651)
Other deciduous broadleafs	74	0.640 (0.187)	0.196 (0.108)	0.049 (0.046)	0.888 (0.278)	50	0.225 (0.107)	1.068 (0.342)
* Acacia*, *Casuarina* and *Eucalyptus*	95	0.618 (0.221)	0.102 (0.098)	0.067 (0.086)	0.790 (0.356)	59	0.175 (0.103)	0.940 (0.404)
Typical evergreen broadleafs	56	0.577 (0.456)	0.158 (0.107)	0.061 (0.065)	0.795 (0.584)	43	0.246 (0.274)	1.071 (0.925)
Other evergreen broadleafs	30	0.675 (0.581)	0.202 (0.156)	0.098 (0.162)	0.975 (0.858)	22	0.229 (0.151)	1.127 (0.689)
Mixed coniferous and broadleaved forest	76	0.481 (0.175)	0.137 (0.084)	0.088 (0.080)	0.707 (0.260)	57	0.172 (0.089)	0.911 (0.363)

* Data are presented as means (standard deviation), and sample sizes (*n*) are also given.

† Forest types (groups) are described in Table S1 in File S2.

### Calculation of BCFs

Here we defined BCFs (in Mg m^−3^) as stand-level ratios for estimating stand-level living biomass (the oven-dried mass per unit area): 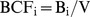
(1)where B_i_ is living biomass (in Mg ha^−1^) of the *i*th tree component (i.e. stem, branch, foliage, and root), aboveground (the sum of all living aboveground tree components) and whole tree (the sum of all living tree components), and V is growing stock volume (in m^3^ ha^−1^). BCFs were developed for tree components (i.e. stem, branch, foliage, and root), as well as aboveground and whole tree biomass. Biomass of dead organic matter (i.e. dead wood and dead branch) and understory vegetation was not included in the calculation of BCFs.

### Statistical analysis

Stands less than 20 years old were only used in analyses for the effects of stand development (expressed by stand age and tree size), but not for other effects (e.g. climatic and edaphic) to minimize the influences of stand age and other unidentifiable factors (e.g. previous management practices and local soil conditions at early growth stages). Differences in BCFs between groups were examined using *t*-test for two groups, or using one-way analysis of variance (ANOVA) followed by Duncan *post hoc* test for more than two groups. Correlation analyses and linear regression were performed to identify the relationships of BCFs with stand development and climatic variables (MAT and MAP). Nonlinear relationships of BCFs with climatic variables were also explored. Using data of older stands (≥20 years), furthermore, multiple regression analyses with backward stepwise procedure were performed to explore the influences of environmental variables (i.e. MAT, MAP and soil fertility) on BCFs. The explanatory variables were selected from MAT, MAP, soil fertility class and interactions between these variables, as well as the quadratic terms of MAT and MAP if they had nonlinear relationships with BCFs. The model selection was based on AIC (Akaike's information criterion) value. A substantial change is considered when a change in AIC of >2 in the performance of the final model over the alternatives [29]. Explanatory power of the model was assessed using the coefficient of determination R^2^.

As ratio data are usually not normally distributed, data of BCFs were log_10_-transformed to normalize the distribution prior to statistical analyses. Statistical significance was determined at *P*≤0.05. Statistical analyses were performed using software SYSTAT version 13.0 (Systat Software Inc., Chicago, Illinois).

## Results

As expected, BCFs exhibited great variation in China's forests (Table 1). Values of BCFs were highest for whole tree and to a lesser extent, above-ground biomass, averaging 0.890 and 0.740 Mg m^−3^, respectively (Table 1). They were much smaller for stems, roots, branches and foliage, averaging 0.505, 0.172, 0.137, and 0.096 Mg m^−3^, respectively. The coefficients of variation (i.e. the ratio of the standard deviation to the mean, in percentage) in these estimates ranged between 49% (for Stem BCF) and 146% (for Foliage BCF).

### Biotic influences on BCFs

Large variations in BCFs existed across forest types (Table 1). The largest variation in BCFs across forest types was in the branch and foliage components, with these varying by 2.7–3.2 times among forest types. The variation in BCFs for other components was only 1.5–1.8 times (Table 1). Also, BCFs varied greatly between functional groups, although not all BCFs differed significantly (Table 2). Coniferous forest had larger Foliage BCFs than broadleaved forest (*P*<0.001), while other BCFs were smaller (*P*<0.001). Compared with evergreen forest, deciduous forest had larger Stem BCFs, Aboveground BCFs, Root BCFs and Whole BCFs (*P*≤0.002), and smaller Foliage BCFs (*P*<0.001), but there were no significant differences in Branch BCFs (*P* = 0.249). Natural forest generally had larger Root BCFs (*P* = 0.030) than planted forest, and smaller Foliage BCFs (*P*<0.001), while other BCFs showed no significant differences (*P*>0.05). When major genera were analyzed separately, *Quercus*, *Castanopsis* and *Phoebe* forests had relatively larger BCFs (except Foliage BCF) than *Cunninghamia*, *Larix*, *Picea* and *Pinus* forests, while *Picea* and *Pinus* forests had larger Foliage BCFs than *Cunninghamia*, *Larix*, *Castanopsis*, *Phoebe* and *Quercus* forests. Among the seven genera, *Cunninghamia* forest had the smallest BCFs.

**Table 2:** Comparison of biomass conversion factors (BCFs, Mg m^−3^) between functional groups *.

Functional group	*n*	Stem BCF	Branch BCF	Foliage BCF	Aboveground BCF	*n*	Root BCF	Whole BCF
Leaf form								
Coniferous forest	365	0.447 (0.121) b	0.107 (0.078) b	0.063 (0.063) a	0.620 (0.216) b	258	0.131 (0.059) b	0.741 (0.251) b
Broadleaved forest	110	0.589 (0.145) a	0.160 (0.087) a	0.036 (0.023) b	0.786 (0.202) a	82	0.195 (0.077) a	0.956 (0.243) a
Leaf lifespan †								
Deciduous forest	117	0.556 (0.152) a	0.126 (0.087) a	0.032 (0.021) b	0.714 (0.215) a	62	0.197 (0.079) a	0.944 (0.254) a
Evergreen forest	376	0.460 (0.131) b	0.116 (0.081) a	0.064 (0.062) a	0.642 (0.224) b	296	0.136 (0.061) b	0.762 (0.256) b
Stand origin								
Natural forest	171	0.499 (0.137) a	0.120 (0.072) a	0.041 (0.029) b	0.661 (0.185) a	105	0.159 (0.067) a	0.820 (0.224) a
Planted forest	336	0.477 (0.147) a	0.119 (0.087) a	0.063 (0.064) a	0.662 (0.243) a	264	0.142 (0.070) b	0.789 (0.285) a
Species genera								
* Cunninghamia*	63	0.355 (0.062) e	0.040 (0.018) e	0.026 (0.014) b	0.422 (0.080) d	55	0.083 (0.020) d	0.511 (0.093) d
* Larix*	56	0.476 (0.102) d	0.068 (0.029) de	0.027 (0.013) b	0.572 (0.110) c	21	0.149 (0.052) c	0.758 (0.173) c
* Picea*	30	0.480 (0.147) cd	0.113 (0.054) bc	0.083 (0.072) a	0.679 (0.212) c	18	0.145 (0.065) c	0.868 (0.280) bc
* Pinus*	182	0.463 (0.121) d	0.133 (0.083) b	0.078 (0.067) a	0.678 (0.218) c	134	0.140 (0.58) c	0.795 (0.239) c
* Castanopsis*	12	0.569 (0.072) b	0.187 (0.076) a	0.038 (0.019) b	0.794 (0.138) b	9	0.186 (0.038) b	0.993 (0.165) b
* Phoebe*	13	0.550 (0.158) bc	0.090 (0.063) cd	0.039 (0.023) b	0.679 (0.216) c	13	0.189 (0.066) b	0.868 (0.261) bc
* Quercus*	22	0.709 (0.166) a	0.179 (0.065) a	0.033 (0.02) b	0.921 (0.191) a	17	0.242 (0.053) a	1.161 (0.140) a

* Data of older stands (≥20 years) were used in this table. Data are presented as means (standard deviation), and sample sizes (*n*) are also given. Different small letters indicate significant (*P*<0.05) differences between functional groups.

† Data for stands dominated jointly by deciduous and evergreen trees were not included in this category.

All BCFs had significant relationships with stand development (Table 3). They decreased significantly (*P*<0.001) with mean DBH, mean tree height and growing stock volume. Moreover, most BCFs decreased with stand age (*P*<0.01). However, increasing trends with stand density were only for Foliage BCFs, Root BCFs and Whole BCFs (*P*<0.001). Among these biometric stand variables, growing stock volume was generally the most powerful variable, which can explain 17.6–54.3% of total variances of BCFs.

**Table 3:** Pearson correlations between biomass conversion factors (BCFs) and stand variables.

BCFs (Mg m^−3^)	Stand age (years)	Mean DBH (cm)	Mean tree height (m)	Stand density (trees ha^−1^)	Growing stock volume (m^3^ ha^−1^)
Stem BCF	−0.043 *ns*	−0.284 *	−0.186 *	−0.058 *ns*	−0.420 *
Branch BCF	−0.109 *	−0.411 *	−0.550 *	−0.007 *ns*	−0.629 *
Foliage BCF	−0.404 *	−0.656 *	−0.789 *	0.311 *	−0.734 *
Aboveground BCF	−0.181 *	−0.498 *	−0.512 *	0.032 *ns*	−0.682 *
Root BCF	−0.238 *	−0.566 *	−0.588 *	0.140 *	−0.697 *
Whole BCF	−0.213 *	−0.599 *	−0.585 *	0.139 *	−0.737 *

All data were log_10_-transformed to linearize relationships between variables and also to reduce the influence of outlying data. *ns*, not significant (*P*>0.05);

*, *P*<0.001.

### Environmental influences on BCFs

**Figure 1:**
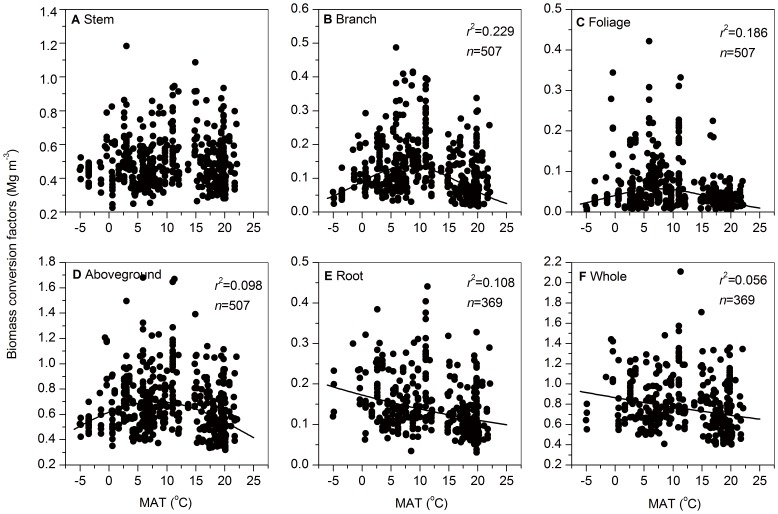
Changes in biomass conversion factors with mean annual temperature (MAT). The regression equations are presented when there are significant relationships between variables (*P*<0.05). Regression equations are given in Table S2 in File S2.

**Figure 2:**
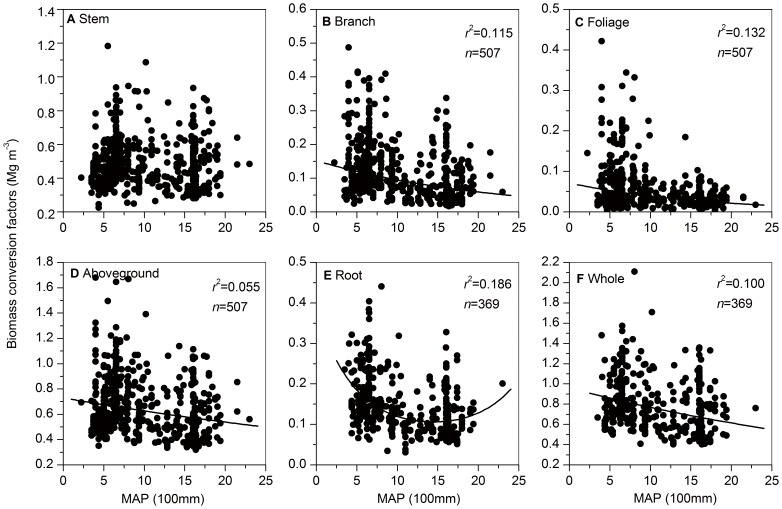
Changes in biomass conversion factors with mean annual precipitation (MAP). The regression equations are presented when there are significant relationships between variables (*P*<0.05). Regression equations are given in Table S2 in File S2.

BCFs except Stem BCF had significant relationships with MAT and MAP (*P*<0.001) (Fig. 1 and 2). Branch BCFs, Foliage BCFs and Aboveground BCFs had humped-shaped relationships with MAT (*P*<0.001) (i.e. they increased with increasing MAT and then declined beyond *ca*. 7.5–8.2°C) (Fig. 1B, 1C and 1D), and they had negatively linear relationship with MAP (*P*<0.001) (Fig. 2B, 2C and 2D). Root BCFs and Whole BCFs were negatively correlated with MAT (*P*<0.001) (Fig. 1E and 1F), but they showed different changes with MAP (Fig. 2E and 2F). Root BCFs showed a U-shaped relationships with MAP (*P*<0.001) (i.e. it decreased and then increased above *ca*. 1400 mm) (Fig. 2E), whereas Whole BCFs did a negatively linear relationship with MAP (*P*<0.001) (Fig. 2F). MAT and MAP explained 5.6–22.9% and 5.5–18.6% of variation in BCFs (except Stem BCFs), respectively (Table 4). When climatic data (MAT and MAP) were combined, they explained 10.0–25.0% of the variation in BCFs (except Stem BCFs) (Table 4), and only MAP had significant effects on both Root BCFs and Whole BCFs (Table S2 in File S2). Furthermore, stronger climatic effects were found on BCFs for functional components (i.e. branch, foliage and root) than BCFs for combined components (i.e. aboveground section and whole trees) (Table 4).

**Table 4:** Explanatory powers (R^2^ values) of final models for the effects of climate and soil fertility on biomass conversion factors (BCFs, Mg m^−3^).

Model	Stem BCF	Branch BCF	Foliage BCF	Aboveground BCF	Root BCF	Whole BCF
MAT	0.000 ns	0.229 **	0.186 **	0.098 **	0.108 **	0.056 **
MAP	0.002 ns	0.115 **	0.132 **	0.055 **	0.186 **	0.100 **
Climate	0.009 ns	0.250 **	0.210 **	0.112 **	0.186 **	0.100 **
Soil fertility class	0.011 ns	0.104 **	0.121 **	0.077 **	0.037 *	0.043 *
Climate+Soil	0.028 ns	0.297 **	0.259 **	0.159 **	0.233 **	0.141 **

Models ‘Climate’ denoted that the explanatory variables were selected by a backward stepwise procedure from mean annual temperature (MAT, °C), mean annual precipitation (MAP, 100 mm) and interactions between these variables, as well as the quadratic terms of MAT and MAP, which had nonlinear relationships with several BCFs (see Fig. 1 and 2). Similarly, models ‘Climate+Soil’ denoted that the explanatory variables were selected by a backward stepwise procedure from MAT, MAP, soil fertility class, interactions between these variables and quadratic terms of continuous variables (MAT and MAP). Details of final models ‘Climate’ and ‘Climate+Soil’ were given in Table S2 and S3 in File S2, respectively. Significance of a model: *ns*, not significant (*P*>0.05);

*, *P*<0.01;

**, *P*<0.001.

**Figure 3:**
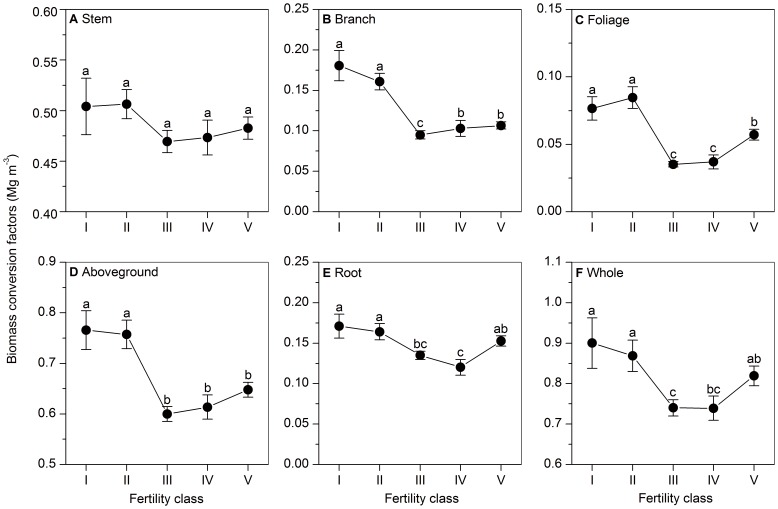
Changes in biomass conversion factors with soil fertility. According to the background values of soil organic matter content, soil fertility is divided into five classes: (I) ≤1.0 g (100 g)^−1^, (II) 1.0–2.0 g (100 g)^−1^, (III) 2.0–3.0 g (100 g)^−1^, (IV) 3.0–4.0 g (100 g)^−1^, and (V) ≥4.0 g (100 g)^−1^. Mean and standard error are shown for each class. Different small letters indicate significant (*P*<0.05) differences between fertility classes.

A general trend for BCFs was observed to decrease and then increase from low to high soil fertility, though Stem BCFs did not differ significantly among fertility classes (*P* = 0.253) (Fig. 3). BCFs except Stem BCF were greater (*P*<0.001) for lower fertility (Class I and II) than for intermediate fertility(Class III or IV), while generally smaller (*P*<0.01) for intermediate fertility than higher fertility (Class IV and V). Moreover, soil fertility class explained only 3.5–12.1% of variation in BCFs (except Stem BCF) (Table 4). When qualitative soil fertility and climatic data (MAT and MAP) were combined, they explained 14.1–29.7% of the variation in in BCFs (except Stem BCF), adding only 4.1–4.9% than climatic data used (Table 4).

## Discussion

### Factors influencing BCFs

Using the statistics (mean, standard deviation and sample size) in Table 1, coefficients of variation (expressed as a percentage) for BCFs were calculated and were in an increasing order: Stem BCF (49%), Whole BCF (55%), Aboveground BCF (56%), Root BCF (73%), Branch BCF (81%) and Foliage BCF (146%). This suggests that BCFs, especially Foliage BCF, Branch BCF and Root BCF, could be sensitive to biotic and environmental variables, and also might have inherent variation in growth rhythm and biomass allocation strategy.

#### (1) Influence of stand development on BCFs

Our study found that BCFs generally decreased with stand age and size (e.g. DBH, tree height and growing stock volume) (Table 3). Similar results for one or more BCFs are observed in specific forest types, e.g. *Betula pubescens* forest [12], *Larix* forest [23], *Picea abies* forest [12], [14], *Pinus densiflora* forest [13], *Pinus sylvestris* forest [12], [15], major forest types of China [11] and temperate broadleaved forest of USA [16].

As trees become larger, stem volume and stem biomass proportion in whole tree biomass increases, meanwhile biomass proportions of other tree compartments decrease proportionally (or remain keep more-or-less unchanged) [30]. In addition, wood density slowly decreases as the trees grow older, although it increases eventually again in older stands as the annual rate of growth abates [14]. Therefore, the trend for BCFs to decrease with stand development appears to be a joint result of changes in the growth rhythm and biomass allocation as stands grow, as well as the definition (i.e. the ratios of biomass to volume) of BCFs.

#### (2) Influence of forest traits on BCFs

Our study found that all BCFs exhibited large variation across forest types (Table 1), while not all of them varied significantly with functional groups (leaf form, leaf lifespan, stand origin, and species genera) (Table 2). Some factors are considered to regulate growth rhythm and biomass allocation strategy of trees in order to accommodate the changes in environments [30]–[33]: photosynthetic rate (PR), relative growth rate (RGR), leaf traits (e.g. leaf form and lifespan), inherent growth rhythm and soil characteristics (e.g. soil water and nutrient availability).

Generally, plants with higher PRs and RGRs show larger stem and root proportions and smaller foliage proportions to whole tree biomass in order to acquire more water and nutrients for achieving similar growth than plants with lower RGRs and PRs [31], [32], [34]. Studies have found that broadleaved trees and deciduous trees generally have higher PRs and RGRs than coniferous and evergreen counterparts [32], [34]. These can explain why broadleaved forest and deciduous forest have larger Stem BCFs and Root BCFs and smaller Foliage BCFs than coniferous forest and evergreen forest, and also why *Quercus*, *Castanopsis* and *Phoebe* forests had relatively larger BCFs (except Foliage BCF).

In addition, plantations are usually established in more productive environments (e.g. more fertile and moister soil) than natural forests, indicating that they can achieve optimal growth with larger foliage proportions and lower root proportions [33], [35]. This could explain why planted forest has larger Foliage BCFs and smaller Root BCFs than natural forest.

#### (3) Influence of environmental factors on BCFs

At broad scales, temperature and precipitation are considered as very important factors in shaping distribution, structure and ecological process of terrestrial biomes, and also influence soil microbial activity and nutrient availability [36]. Luo et al. [37] found that higher temperature and precipitation favor higher tree growth and more photosynthate allocated to tree stem, suggesting that the size and biomass proportion of stems have positive relationships with MAT and MAP. This promotes the defensive capacity of trees against environmental perturbation, and might contribute to ensure their better soil-to-leaf hydraulic conductance [38], [39].

Our study found that relationships of BCFs with MAT and MAP varied with tree components (e.g. branch, foliage, and root) (Figs. 1 and 2). Branch BCFs, Foliage BCFs and Aboveground BCFs decreased with MAP (Fig. 2), which can also be explained by higher tree growth and light use efficiency, and hence decreasing biomass proportions of photosynthetic compartments (i.e. foliage and branch) with increasing MAP. However, a humped-shaped change of BCFs with increasing MAT was observed for three BCFs (i.e. Branch BCFs, Foliage BCFs and Aboveground BCFs) (Fig. 1), which can be explained by the following causes. At lower temperatures, increasing temperature favors tree growth by better growth conditions (e.g. higher soil microbial activity and nutrient availability), and thus results in more branches and leaves for more water and photosynthate at a tree size; at higher temperatures, larger transpiration and respiration lead to a reduction in biomass allocation to branches and leaves to minimize maintenance costs at a tree size. The decreasing trend of Root BCFs with MAT (Fig. 1) can be attributed to higher tree growth and lower root biomass proportion with increasing MAT [37], and the effect of MAT might be indirect because the effect disappeared when both MAT and MAP were considered (Table S2 in File S2). Root BCFs had a U-shaped pattern with increasing MAP across China's forests (Fig. 2). With increasing precipitation, water supply and soil fertility becomes abundant (Fig. S2 in File S2), and thus tree growth will be accelerated and trees can allocate less biomass to roots to absorb soil water and nutrients. However, when precipitation exceeds the demand of trees, tree growth will be inhibited by soil nutrient availability rather than water (Fig. S2 in File S2), resulting in more biomass would be allocated into roots to absorb more nutrients from relatively infertile soil with high precipitation [37].

Terrestrial plants take up most of their nutrients and water directly from soils. Soil chemical attributes (e.g. pH and mineral nutrient availability) are critical to plant growth and thus affect biomass allocation patterns [33], [40], [41]. Moreover, climate influences soil chemical attributes and thus shapes the vegetation biogeography [36]. Across China's forests, MAT and MAP showed a humped-shaped change with increasing soil fertility (Fig. S2 in File S2). As soil fertility increases, soil nutrients become abundant and thus trees show rapider growth in the size and broader tree rings. That might lead to smaller wood density and less foliage and root proportions in tree biomass, which can explain a decreasing trend in BCFs with increasing soil fertility at lower soil fertility. However, when soil nutrients exceed the demand of trees, tree growth will be inhibited by temperature and precipitation, and trees show slower growth in the size and narrower tree rings. That might lead to larger wood density and more foliage and root proportions in tree biomass, which can explain an increasing trend in BCFs with increasing soil fertility at higher fertility. In addition, soil fertility showed only a weak effect on BCFs, which add only 4.1–4.9% in explaining the variation of BCFs meanwhile climatic data (MAT and MAP) were considered (Table 4). And the effect of soil fertility was reflected by the interactions between soil fertility and climate (MAT and MAP) (Table S3 in File S2).

Apart from the above mentioned influencing factors, management practices (e.g. thinning, pruning and fertilization) might lead to changes in growth rhythm and biomass allocation of trees, and thus affect BCFs. In this study, however, we did not analyze effects of management practices, largely due to the lack of management descriptions in original data sources.

### Implications for biomass and carbon accounting

Our study presented forest type-specific values of various BCFs in Table 1. When comparing our values for BCFs with others available [2], means of Aboveground BCFs for China's forests and for most forest types (Table 1) were smaller than IPCC default values (mean = 1.405 Mg m^−3^; range = 0.692–2.913 Mg m^−3^) [2], indicating that a large overestimation would result if IPCC default values was used in the estimation of China's forest carbon storage. In addition, China conducted seven consecutive national forest inventories between 1973 and 2008, which can provide statistical data of forest area and growing stock volume by age class and forest type [17]. To be compatible with these FIDs, forests were categorized by tree species, growing region and stand origin into five age classes (young, middle-aged, premature, mature and overmature) (Table S4 in File S2), and then forest type-specific BCFs by age class were given in Table S5–S10 in File S2. However, the use of the values of BCFs for premature, mature and overmature forests might induce somewhat uncertainty in forest biomass and carbon estimates, due to limited data for the three growth stages.

Considering large coefficients of variation for BCFs, the use of constant values of BCFs, especially Foliage BCF, Branch BCF and Root BCF, would lead to high uncertainties in the estimates of forest biomass and carbon. Our results showed that high variation in BCFs were related to forest types (Table 1), functional groups (leaf form, leaf lifespan, stand origin and species genera) (Table 2), stand development (Table 3), climate (MAT and MAP) (Fig. 1 and 2) and soil fertility (Fig. 3). In order to reduce uncertainties induced by BCFs, we recommend that: (i) different values of BCFs should be selected for forest types and functional groups, and (ii) climatic and edaphic factors, especially climatic factors, should be considered in developing predictive models of BCFs (except Stem BCF). To further reduce uncertainty induced by BCFs in forest carbon estimates, future emphases should be placed on obtaining BCF estimates for region and forest types where there is currently little data available, and also on developing the predictive models of BCFs (except Stem BCF) for a specified forest type integrating environmental factors, especially climatic factors (MAT and MAP). Certainly, if the predictive model of a certain BCF integrating stand (e.g. growing stock volume) and environmental factors (e.g. MAT, MAP, and soil fertility) has poor performance (e.g. low R^2^), it is unsuitable for the model to estimate values of the BCF which are used to calculate forest biomass and carbon stock.

## Conclusions

BCFs are considered as important parameters in quantifying forest biomass carbon stock and dynamics. Knowledge of the sources of variation in BCFs is prerequisite to accurately determining the values of BCFs, thereby reducing the uncertainties in forest biomass carbon estimates. However, prior to this study there has been little information on possible causes of the variation in BCFs, especially environmental effects.

Our study was the first to give China's forest type-specific values of BCFs, and to examine the variation in BCFs in relation to forest type, stand development, climate and soil fertility. Results showed that BCFs generally varied significantly with forest types, stand development (stand age and size), climate (MAT and MAP) and soil fertility (fertility class). These results indicate that: (i) different values of BCFs should be selected for forest types and functional groups, and (ii) climatic and edaphic factors, especially climatic factors, should be considered in developing predictive models of BCFs (except Stem BCF).

## Supporting Information

File S1 **Description of forest biomass dataset of China.** (DOC)

File S2 **It included 2 figures and 10 tables.** (DOC). **Figure S1** Spatial distribution of study sites used in this study; **Figure S2** Relationships between soil fertility and climate: (A) mean annual temperature (MAT) and (B) mean annual precipitation (MAP); **Table S1** Categorization of forest type by dominant species and ecogeographical zone; **Table S2** Summary of multiple regression equations for the effects of climate on biomass conversion factors (BCFs, Mg m^−3^); **Table S3** Summary of final models for the effects of climate and soil fertility on biomass conversion factors (BCFs, Mg m^−3^); **Table S4** Categorization of age class by tree species, growing region and stand origin; **Table S5** Statistics of stem biomass conversion factors (Mg m^−3^) by forest type and age class; **Table S6** Statistics of branch biomass conversion factors (Mg m^−3^) by forest type and age class; **Table S7** Statistics of foliage biomass conversion factors (Mg m^−3^) by forest type and age class; **Table S8** Statistics of aboveground biomass conversion factors (Mg m^−3^) by forest type and age class; **Table S9** Statistics of root biomass conversion factors (Mg m^−3^) by forest type and age class; **Table S10** Statistics of whole biomass conversion factors (Mg m^−3^) by forest type and age class.
(DOC)
