# Supplementary material for: Dissecting Variation in Biomass Conversion Factors across China’s Forests: Implications for Biomass and Carbon Accounting
Source: PLoS One. 2014 Apr 11;9(4):e94777. doi: 10.1371/journal.pone.0094777 (PMC3984257; doi:10.1371/journal.pone.0094777)
Supplement: File S1 — Description of forest biomass dataset of China. (DOC) [file pone.0094777.s001.doc]

**File S1** Description of forest biomass dataset of China

To date, we have reviewed all available published studies and previous datasets [1-3] on forest biomass and its allocation of China, and compiled a harmonized and quality-checked forest biomass and its allocation dataset of China (excluding Hong Kong, Macao and Taiwan). These published studies between 1978 and 2008 were retrieved from famous libraries (National Library of China, and Forestry Library of China) and online full-text databases (China Knowledge Resource Integrated Database, China Science and Technology Journal Database, and Wanfang Data).

Using the following criteria, all available studies were critically assessed to obtain reliable biomass data. (1) Forests, which were strongly influenced by recent disturbances and/or grown in specific sites, were excluded: recently disturbed stands (by pruning, thinning, coppicing, fire, insect pest, etc.), economic stands (for producing fruits, edible oils, medicinal herbs, industrial raw materials, etc.), agroforestry systems (integrating with crops and/or animals), wetland forests (swamp forest, peatland forest and intertidal forest), and other stands grown in atypical sites (e.g. urban, desert, riverine, timberline, and heavily eroded sites). (2) Sampling and measuring protocols (or procedures) for determining the oven-dried mass of living tree components (stem, branch, foliage, root, etc.) should be explicitly described in original studies, including suitable survey time (generally during growing season, especially for deciduous forest), sample plot setting, determination methods for fresh mass of tree components, and stand biomass estimation methods [1]. (3) The tree-level biomass (the oven-dried mass) of sample trees was measured using destructive harvesting and weighing, and then scaled up to stand level (the oven-dried mass per unit area) using clear-cutting method, (stratified) average tree method and allometric equation method. For a study site, allometric equations with one or more stand variables (e.g. diameter at breast height (1.3m) (DBH), tree height or their combinations) as predictor variables were developed based on biomass measurements of individual trees, and were then used to estimate stand-level biomass within the study site. It should be noted that those studies were excluded, where biomass data were estimated using previous models (or equations) or excluded some component biomass (e.g. branch and foliage). (4) If biomass data of tree roots were available in studies, they should be the biomass of the whole root systems (including root crown and fine roots). Moreover, root biomass should be measured using full-excavation method to cover enough area (equal to or larger than the average area the sample trees covered) and to reach the maximum root depth, or be supplemented by soil pit method for fine roots. (5) Each biomass measurement was checked for duplicate and suspicious data. Those measured data more (or less) than twice ones in similar growth stage and site condition were considered as suspicious data. These suspicious data were then re-checked by expert judgment in order to decide whether they can be included in our dataset.

As a result, we collected 516 ones from thousands of available studies, and compiled a comprehensive forest biomass dataset of China. The dataset consisted of 1,607 entries on the biomass (the oven-dried mass per unit area, Mg ha^-1^) of tree components (stem, branch, foliage, root, etc.). Associated information if available was also collected, including stand description (forest type, dominant tree species, stand origin, stand age (years), mean DBH (cm), mean tree height (m), stand density (trees ha^-1^), growing stock volume (m^3^ ha^-1^)), geographical location (latitude (°), longitude (°) and altitude (m)), and climate (mean annual temperature (MAT, °C) and mean annual precipitation (MAP, mm)), and soil organic matter content (SOM, g (100g)^-1^) of topsoil layer (0-20cm). However, not all original studies reported growing stock volume, geographical location, climatic data (MAT and MAP), and SOM. These missing variables were estimated as follows:

(1) Growing stock volume

Growing stock volumes were obtained for those stands without actual measurements but with adequate information available (mean DBH, mean tree height and stand density). Stands with actual measurements of growing stock volume (GSV), mean DBH, mean tree height (H) and stand density (D) were used to establish relationships between observed (GSV_obs_) and estimated (GSV_est_) growing stock volumes. GSV_est_ was calculated by multiplying mean stem volume, where mean stem volume was calculated using a conical shape model [4], with stand density (D), i.e. GSV_est_=1/3·π·(DBH/2)^2^·H·D. A simple correction factor, *f*, was obtained as the slope of the regression line through the origin (GSV_obs_=GSV_est_·*f*). Highly significant (*P*<0.001) regression lines with high *r*^2^ were obtained for forest types (groups) (Table S1). Finally, forest type-specific values of *f* were used to adjust estimated growing stock volume for those stands without observed ones but with adequate information available.

**Table S1** Relationships between observed (GSV_obs_) and estimated (GSV_est_) growing stock volume for forest types (groups) in China. The relationship is expressed as GSV_obs_=GSV_est_·*f*. Symbol *f* is a simple correction factor, SE is standard error of the factor *f*, *n* is sample size, *r*^2^ is the coefﬁcient of determination, and SEE is standard error of the estimate.

| Forest type (group) | *f* (SE) | *n* | *r*^2^ | SEE |
| --- | --- | --- | --- | --- |
| *Abies* and *Picea* | 1.421 (0.058) | 13 | 0.830 | 35.322 |
| *Cunninghamia lanceolata* | 1.534 (0.009) | 171 | 0.987 | 20.006 |
| *Cupressus* and *Fokienia* | 1.511 (0.022) | 16 | 0.992 | 10.843 |
| *Larix* | 1.401 (0.026) | 50 | 0.931 | 22.102 |
| *Pinus koraiensis* | 1.416 (0.035) | 22 | 0.932 | 29.689 |
| *P. massoniana* | 1.425 (0.020) | 51 | 0.977 | 22.316 |
| *P. tabuliformis* | 2.018 (0.033) | 32 | 0.973 | 7.906 |
| Other pines | 1.500 (0.027) | 32 | 0.979 | 15.121 |
| Other conifers | 1.657 (0.054) | 22 | 0.940 | 38.806 |
| Deciduous broadleaved forest | 1.442 (0.056) | 29 | 0.880 | 25.485 |
| *Acacia*, *Casuarina* and *Eucalyptus* | 1.385 (0.025) | 43 | 0.974 | 21.303 |
| Other evergreen broadleaved forests | 1.531 (0.025) | 53 | 0.956 | 25.799 |
| Coniferous-broadleaved mixed forest | 1.443 (0.044) | 44 | 0.897 | 34.637 |

(2) Geographical location

For study sites without geographical locations in original studies, these missing data (i.e. latitude, longitude and altitude) were estimated using the geographical center of the site from Google Earth ^TM^.

(3) Climatic data

Missing climatic data (MAT and MAP) were extracted using geographical coordinates from a 30 arc-seconds (*ca*. 1 km at the equator) resolution global climate database (WorldClim) ([http://www.worldclim.org](http://www.worldclim.org/)) [5]. The accuracy of estimated climatic data was assessed by comparing estimated data with actual data, for those sites where actual data were given in original studies. Figure S1 shows that, coefficients of determination (*r*^2^) between the estimated and actual values were 0.992 for MAT and 0.967 for MAP; and the slope was 1.004 and 0.964, respectively. This suggests that using WorldClim to estimate climatic data is feasible in our dataset.


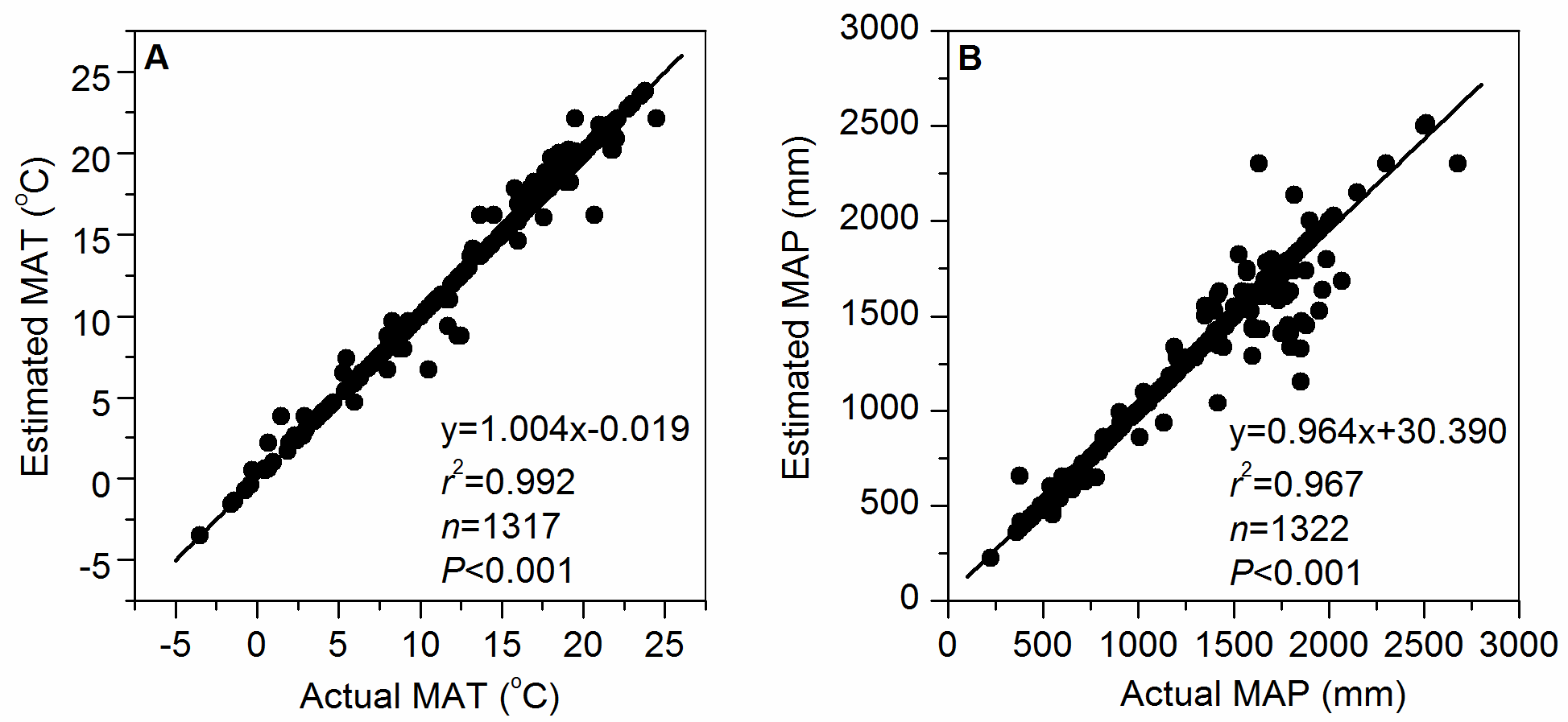


**Figure S1** Relationships between actual and estimated values for (A) mean annual temperature (MAT) and (B) mean annual precipitation (MAP). Solid lines denote the regression lines.

(4) Soil fertility

To determine soil fertility classes based on SOM background values, SOM data were extracted using geographical coordinates from a 30 arc-seconds resolution soil database of China (<http://globalchange.bnu.edu.cn/research/soil2>) [6].

**References**

1. Feng ZW, Wang XK, Wu G (1999) Biomass and productivity of forest ecosystems in China. Beijing: Science Press. 241pp.
2. Luo TX (1996) Patterns of net primary productivity for Chinese major forest types and their mathematical models (Ph.D. dissertation). Beijing: Commission for Integrated Survey of Natural Resources, Chinese Academy of Sciences. 211pp.
3. Wang XP, Fang JY, Zhu B (2008) Forest biomass and root-shoot allocation in northeast China. Forest Ecology and Management 255: 4007-4020.
4. West PW (2009) Tree and forest measurement, 2nd Edition. Berlin: Springer-Verlag. 190pp.
5. Hijmans RJ, Cameron SE, Parra JL, Jones PG, Jarvis A (2005) Very high resolution interpolated climate surfaces for global land areas. International Journal of Climatology 25: 1965-1978.
6. Shangguan W, Dai YJ, Liu BY, Zhu AX, Duan QY, et al. (2013) A China data set of soil properties for land surface modeling. Journal of Advances in Modeling Earth Systems 5: 1-13.
