# Supplementary material for: Dissecting Variation in Biomass Conversion Factors across China’s Forests: Implications for Biomass and Carbon Accounting
Source: PLoS One. 2014 Apr 11;9(4):e94777. doi: 10.1371/journal.pone.0094777 (PMC3984257; doi:10.1371/journal.pone.0094777)
Supplement: File S2 — It included 2 figures and 10 tables. (DOC). Figure S1 Spatial distribution of study sites used in this study; Figure S2 Relationships between soil fertility and climate: (A) mean annual temperature (MAT) and (B) mean annual precipitation (MAP); Table S1 Categorization of forest type by dominant species and ecogeographical zone; Table S2 Summary of multiple regression equations for the effects of climate on biomass conversion factors (BCFs, Mg m−3); Table S3 Summary of final models for the effects of climate and soil fertility on biomass conversion factors (BCFs, Mg m−3); Table S4 Categorization of age class by tree species, growing region and stand origin; Table S5 Statistics of stem biomass conversion factors (Mg m−3) by forest type and age class; Table S6 Statistics of branch biomass conversion factors (Mg m−3) by forest type and age class; Table S7 Statistics of foliage biomass conversion factors (Mg m−3) by forest type and age class; Table S8 Statistics of aboveground biomass conversion factors (Mg m−3) by forest type and age class; Table S9 Statistics of root biomass conversion factors (Mg m−3) by forest type and age class; Table S10 Statistics of whole biomass conversion factors (Mg m−3) by forest type and age class. (DOC) [file pone.0094777.s002.doc]

**File S2** This file included 2 figures and 10 tables.

**Figures**


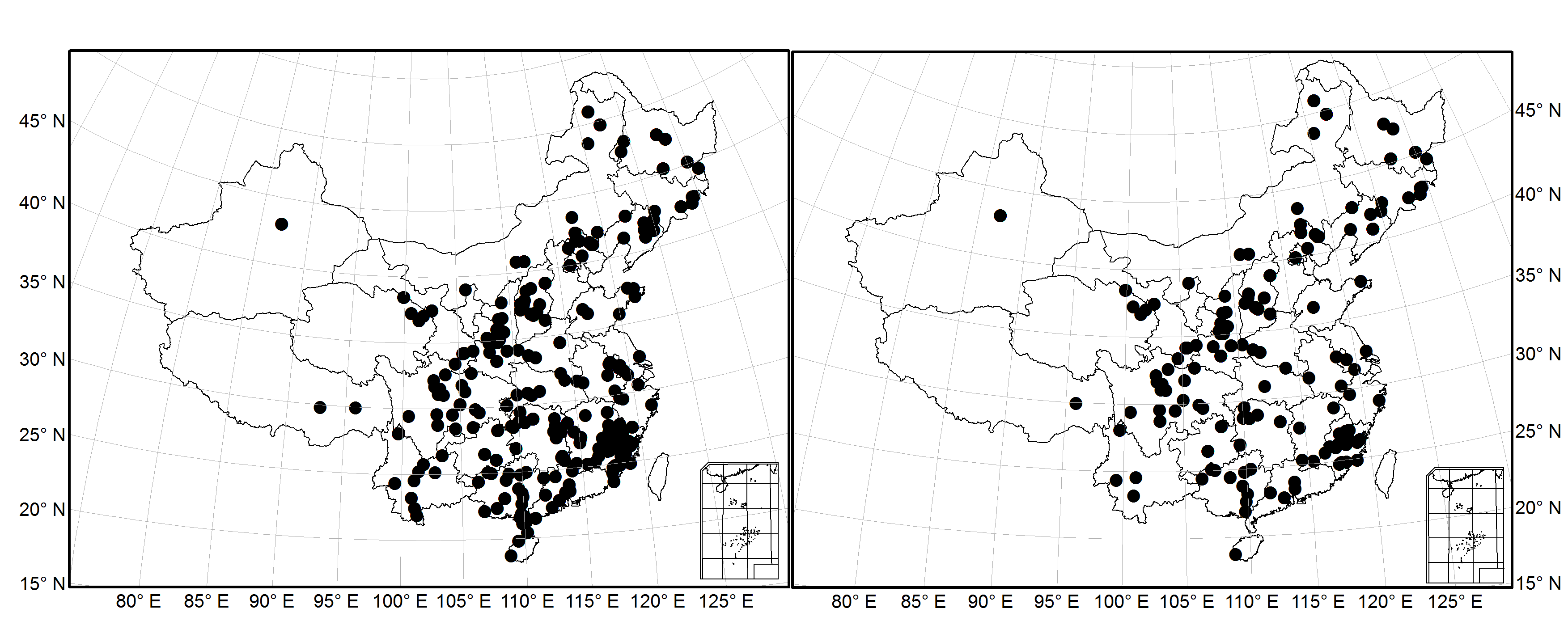


**Figure S1** Spatial distribution of study sites used in this study. The distributions of sites (black dots) are presented for (Left) total data and (Right) older stands (≥20 years). Province boundaries are also shown.


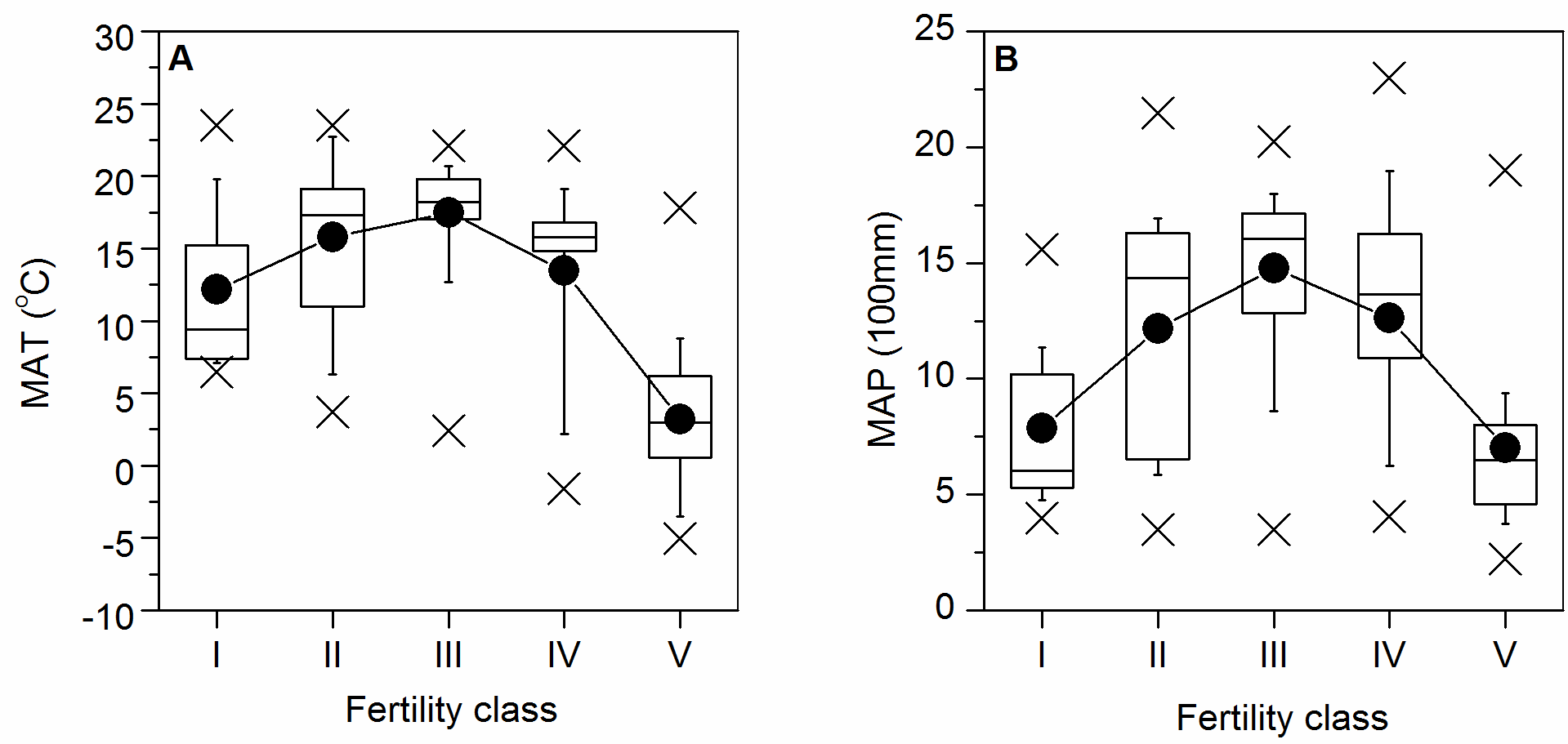


**Figure S2** Relationships between soil fertility and climate: (A) mean annual temperature (MAT) and (B) mean annual precipitation (MAP). According to the background values of soil organic matter content, soil fertility is divided into five classes: (I) ≤1.0 g/100g, (II) 1.0-2.0 g/100g, (III) 2.0-3.0 g/100g, (IV) 3.0-4.0 g/100g and (V) ≥4.0 g/100g. Mean (black dot), median (solid line), lower quartile (25%), upper quartile (75%), minimum and maximum are shown. The boundary of the box closest to zero indicates lower quartile, and the boundary of the box farthest from zero does upper quartile. The whiskers above and below the box indicate the 90th and 10th percentiles.

**Tables**

**Table S1** Categorization of forest type by dominant species and ecogeographical zone

| Forest type (group) | Forest types included |
| --- | --- |
| *Abies* and *Picea* | Forests dominated by *Abies fabri* and *Picea* sp. (*P. asperata*, *P. brachytyla* var. *complanata*, *P. crassifolia*, *Picea koraiensis*, *P. likiangensis* var. *balfouriana*, *P. likiangensis* var. *linzhiensis*, *P. schrenkiana* and *P. wilsonii*), and mixed *Abies*-*Picea* stands. |
| *Cunninghamia lanceolata* | Forest dominated by *Cunninghamia lanceolata*. |
| *Cupressus* and *Fokienia* | Forests dominated by *Cupressus funebris* and *Fokienia hodginsii*. |
| *Larix* | Forests dominated by *Larix* sp. (*L.* *gmelinii*, *L. kaempferi*, *L. mastersiana*, *L. olgensis* and *L. principis-rupprechtii*) |
| *Pinus koraiensis* | Forest dominated by *Pinus koraiensis*. |
| *P. massoniana* | Forest dominated by *Pinus massoniana*. |
| *P. tabuliformis* | Forest dominated by *Pinus tabuliformis*. |
| Other temperate pines and conifers | Forests dominated by temperate *Pinus* sp. (*P. armandii*, *P. densata*, *P. densiflora*, *P. sylvestris* var. *mongolica*, *P. sylvestris* var. *sylvestriformis* and *P. thunbergii*), *Platycladus orientalis* and *Sabina przewalskii*, and mixed coniferous stands in temperate zone. |
| Other subtropical pines and conifers | Forests dominated by *Cryptometria* sp. (*C. fortunei* and *C. japonica*), *Keteleeria davidiana*, *Metasequoia glyptostroboides*, subtropical *Pinus* sp. (*P. elliottii*, *P. fenzeliana*, *P. kesiya* var. *langbianensis*, *P. rigida* var. *serotina,* *P. taeda* and *P. taiwanensis*) and *Taiwania flousiana*, and mixed coniferous forests in subtropical zone. |
| *Betula* and *Populus* | Forests dominated by *Betula* sp. (*B. albosinensis*, *B. alnoides*, *B. platyphylla*, *B. luminifera*,) and *Populus* sp. (e.g. *P. canadensis*, *P. davidiana*, *P. simonii*, and clones), and mixed *Betula*-*Populus* stands. |
| *O*ther deciduous broadleafs | Forests dominated by *Acer truncatum*, *Alniphyllum fortune*, *Alnus* sp. (*A. cremastogyne* and *A. formosana*), *Choerospondias axillaris*, *Fraxinus mandshurica*, *Liquidambar formosana*, *Liriodendron chinensis*, *Paulownia fortune*, deciduous *Quercus* sp. (*Q. acutissima*, *Q. aliena* var. *acutiserrata*, *Q. mongolica*, *Q. variabilis* and *Q. wutaishanica*), *Robinia pseudoacacia, Sassafras tzumu* and *Zenia insignis*,, and mixed deciduous broadleaved forests. |
| *Acacia*, *Casuarina* and *Eucalyptus* | Forests dominated by *Acacia* sp. (*A. auriculiformis*, *A. cincinnata*, *A. crassicarpa*, *A. dealbata*, *A. leptocarpa*, *A. mangium*, *A. melanoxylon*, etc.), *Casuarina* sp. (*C. equisetifolia* and *C. junghuhniana*) and *Eucalyptus* sp. (*E. camaldulensis*, *E. citriodora*, *E. dunnii*, *E. exserta*, *E. globulus*, *E. maculata*, *E. saligna*, *E. tereticornis*, etc.), and their mixed stands. |
| Typical evergreen broadleafs | Forests dominated by *Castanopsis* sp. (e.g. *C.* *carlesii*, *C. fargesii*, *C. fissa*, *C. fordii*, *C. hystrix*, *C. kawakamii*, *C. lamonteii*), *Cinnamomum camphora*, *Cyclobalanopsis* sp., *Lithocarpus* sp., *Machilus pauhoi*, *Phoebe bournei* and *Schima superba*. |
| Other evergreen broadleafs | Forests dominated by *Altingia gracilipes*, *Elaeocarpus sylvestris*, *Gordonia axillaris*, *Macaranga deheiculata*, *Mallotus paniculatus*, *Manglietia* sp. (*M. hainanensis* and *M. yuyuanensis*), *Michelia macclurei*, *Mytilaria laosensis*, *Ormosia* sp. (*O. hosiei* and *O. xylocarpa*), *Quercus pannosa*, *Sloanea sinensis*, *Trema tomentosa* and *Tsoongiodendron odorum*, and mixed evergreen broadleaved forests in subtropical and tropical zones. |
| Mixed coniferous and broadleaved forest | Forests dominated by both conifers (e.g. *Cunninghamia lanceolata*, *Cupressus funebris*, *Fokienia hodginsii*, *Larix* *gmelinii*, *L. kaempferi*, *Pinus koraiensis*, *P. massoniana*, *P. elliottii* and *P. tabuliformis*) and broadleaves (e.g. *Acer truncatum*, *Alnus tinctoria*, *Castanopsis* sp., *Elaeocarpus sylvestris*, *Phellodendron amurense*, *Quercus variabilis, Sassafras tzumu*, *Schima superba*). |

**Table S2** Summary of multiple regression equations for the effects of climate on biomass conversion factors (BCFs, Mg m^-3^). BCFs were log_10_-transformed. ‘Climate’ denoted that the explanatory variables were selected by a backward stepwise procedure from mean annual temperature (MAT, ^o^C), mean annual precipitation (MAP, 100 mm) and interactions between these variables, as well as the quadratic terms of MAT and MAP, which had nonlinear relationships with several BCFs.

| BCFs (*y*) | *x* | Equation | *R*^2^ | *P*-value |
| --- | --- | --- | --- | --- |
| Stem BCF | MAT |  |  | >0.05 |
|  | MAP |  |  | >0.05 |
|  | Climate |  |  | >0.05 |
| Branch BCF | MAT | *y*=-1.0435+0.0430•MAT-0.0026•(MAT)^2^ | 0.229 | <0.001 |
|  | MAP | *y*=-0.8184-0.0205•MAP | 0.115 | <0.001 |
|  | Climate | *y*=-0.9481+0.0446•MAT-0.0021•(MAT)^2^-0.0191•MAP | 0.250 | <0.001 |
| Foliage BCF | MAT | *y*=-1.3864+0.0381•MAT-0.0025•(MAT)^2^ | 0.186 | <0.001 |
|  | MAP | *y*=-1.1455-0.0262•MAP | 0.132 | <0.001 |
|  | Climate | *y*=-1.2625+0.0401•MAT-0.0019•(MAT)^2^-0.0248•MAP | 0.210 | <0.001 |
| Aboveground  BCF | MAT | *y*=-0.2088+0.0131•MAT-0.0008•(MAT)^2^ | 0.098 | <0.001 |
|  | MAP | *y*=-0.1370-0.0066•MAP | 0.055 | <0.001 |
|  | Climate | *y*=-0.1717+0.0137•MAT-0.0006•(MAT)^2^-0.0074•MAP | 0.112 | <0.001 |
| Root BCF | MAT | *y*=-0.7626-0.0096•MAT | 0.108 | <0.001 |
|  | MAP | *y*=-0.4110-0.0780•MAP+0.0027•(MAP)^2^ | 0.186 | <0.001 |
|  | Climate | *y*=-0.4110-0.0780•MAP+0.0027•(MAP)^2^ | 0.186 | <0.001 |
| Whole BCF | MAT | *y*=-0.0625-0.0049•MAT | 0.056 | <0.001 |
|  | MAP | *y*=-0.0172-0.0097•MAP | 0.100 | <0.001 |
|  | Climate | *y*=-0.0172-0.0097•MAP | 0.056 | <0.001 |

**Table S3** Summary of final models for the effects of climate and soil fertility on biomass conversion factors (BCFs, Mg m^-3^)

| Factors | Coeff. | d.f. | MS | *F*-ratio | *P*-value |  | Factors | Coeff. | d.f. | MS | *F*-ratio | *P*-value |
| --- | --- | --- | --- | --- | --- | --- | --- | --- | --- | --- | --- | --- |
| Branch BCF (Model R^2^=0.297; AIC value=62.9) | | | | | |  | Foliage BCF (Model R^2^=0.259; AIC value=264.6) | | | | | |
| MAT | 0.044 | 1 | 1.970 | 30.6 | <0.001 |  | MAT | 0.042 | 1 | 1.843 | 19.2 | <0.001 |
| (MAT)^2^ | -0.004 | 1 | 2.434 | 37.8 | <0.001 |  | (MAT)^2^ | -0.004 | 1 | 2.126 | 22.2 | <0.001 |
| MAP | -0.050 | 1 | 0.594 | 9.2 | 0.003 |  | MAP | -0.065 | 1 | 0.994 | 10.4 | 0.001 |
| MAT×MAP | 0.004 | 1 | 0.725 | 11.3 | 0.001 |  | MAT×MAP | 0.004 | 1 | 0.808 | 8.4 | 0.004 |
| Fertility×MAT |  | 4 | 0.418 | 6.5 | <0.001 |  | Fertility×MAT |  | 4 | 0.480 | 5.0 | 0.001 |
| Fertility×MAP |  | 4 | 0.371 | 5.8 | <0.001 |  | Fertility×MAP |  | 4 | 0.463 | 4.8 | 0.001 |
| Residual |  | 494 | 0.064 |  |  |  | Residual |  | 494 | 0.096 |  |  |
| Aboveground BCF (Model R^2^=0.159; AIC value=-622.6) | | | | | |  | Root BCF (Model R^2^=0.233; AIC value=-219.6) | | | | | |
| MAT | 0.016 | 1 | 0.278 | 16.7 | <0.001 |  | MAP | -0.086 | 1 | 1.057 | 33.9 | <0.001 |
| (MAT)^2^ | -0.001 | 1 | 0.283 | 17.0 | <0.001 |  | (MAP)^2^ | 0.003 | 1 | 0.695 | 22.3 | <0.001 |
| MAP | -0.020 | 1 | 0.097 | 5.8 | 0.016 |  | Fertility×MAT |  | 4 | 0.132 | 4.2 | 0.002 |
| MAT×MAP | 0.002 | 1 | 0.105 | 6.3 | 0.012 |  | Fertility×MAP |  | 4 | 0.117 | 3.7 | 0.005 |
| Fertility×MAT |  | 4 | 0.094 | 5.6 | <0.001 |  | Residual |  | 358 | 0.031 |  |  |
| Fertility×MAP |  | 4 | 0.080 | 4.8 | 0.001 |  |  |  |  |  |  |  |
| Residual |  | 494 | 0.017 |  |  |  |  |  |  |  |  |  |
| Whole BCF (Model R^2^=0.141; AIC value=-433.7) | | | | | |  |  |  |  |  |  |  |
| MAT×MAP | -0.0003 | 1 | 1.052 | 7.3 | 0.007 |  |  |  |  |  |  |  |
| Fertility×MAT |  | 4 | 0.089 | 4.1 | 0.003 |  |  |  |  |  |  |  |
| Fertility×MAP |  | 4 | 0.098 | 4.3 | 0.002 |  |  |  |  |  |  |  |
| Residual |  | 259 | 0.033 |  |  |  |  |  |  |  |  |  |

Explanatory variables used include mean annual temperature (MAT, ^o^C), mean annual precipitation (MAP, 100 mm), soil fertility class (Fertility) and interactions between these variables, as well as the quadratic terms of MAT and MAP, which had nonlinear relationships with several BCFs (see Table S2 in File S2). The ﬁnal models were obtained by a backward stepwise procedure. All explanatory variables were excluded from final models of Stem BCF, and thus model results were not presented. Abbreviations: Coeff., coefficient (Soil fertility class was category variables and thus coefficient for each category were not shown); AIC, Akaike’s information criterion; d.f., degree of freedom; MS, mean squares.

**Table S4** Categorization of age class by tree species, growing region and stand origin *

| Dominant tree species | Region † | Origin ‡ | Age class (yr.) | | | | |
| --- | --- | --- | --- | --- | --- | --- | --- |
|  |  |  | Young | Middle-aged | Premature | Mature | Overmature |
| *Cupressus* sp., *Picea* sp., *Pinus koraiensis*, *Taxus* sp., *Tsuga* sp. | N | NF | ≤60 | 61-100 | 101-120 | 120-160 | ≥161 |
|  | N | PF | ≤40 | 41-60 | 61-80 | 81-120 | ≥121 |
|  | S | NF | ≤40 | 41-60 | 61-80 | 81-120 | ≥121 |
|  | S | PF | ≤20 | 21-40 | 41-60 | 61-80 | ≥81 |
| *Abies* sp., *Larix* sp., *Pinus densiflora*, *P. sylvestris* var. *mongolica*, *P. thunbergii* | N | NF | ≤40 | 41-80 | 81-100 | 101-140 | ≥141 |
|  | N | PF | ≤20 | 21-30 | 31-40 | 41-60 | ≥61 |
|  | S | NF | ≤40 | 41-60 | 61-80 | 81-120 | ≥121 |
|  | S | PF | ≤20 | 21-30 | 31-40 | 41-60 | ≥61 |
| *Pinus armandii*, *P. densata, P. kesiya* var. *langbianensis*, *P. massoniana*, *P. tabulaeformis*, *P. yunnanensis* | N | NF | ≤30 | 31-50 | 51-60 | 61-80 | ≥81 |
|  | N | PF | ≤20 | 21-30 | 31-40 | 41-60 | ≥61 |
|  | S | NF | ≤20 | 21-30 | 31-40 | 41-60 | ≥61 |
|  | S | PF | ≤10 | 11-20 | 21-30 | 31-50 | ≥51 |
| *Cryptomeria* sp., *Cunninghamia* sp., *Metasequoia* sp. | S | PF | ≤10 | 11-20 | 21-25 | 26-35 | ≥36 |
| *Acacia* sp., *Casuarina* sp., *Eucalyptus* sp., *Melia* sp., *Paulownia* sp., *Populus* sp., *Pterocarya* sp., *Salix* sp., *Sassafras* sp., etc. | N | PF | ≤10 | 11-15 | 16-20 | 21-30 | ≥31 |
|  | S | PF | ≤5 | 6-10 | 11-15 | 16-25 | ≥26 |
| *Betula* sp., *Davidia* sp., *Liquidambar* sp., *Schima* sp., *Ulmus* sp. | N | NF | ≤30 | 31-50 | 51-60 | 61-80 | ≥81 |
|  | N | PF | ≤20 | 21-30 | 31-40 | 41-60 | ≥61 |
|  | S | NF | ≤20 | 21-40 | 41-50 | 51-70 | ≥71 |
|  | S | PF | ≤10 | 11-20 | 21-30 | 31-50 | ≥51 |
| *Castanopsis* sp., *Cinnamomum* sp., *Fraxinus* sp., *Juglans* sp., *Machilus* sp., *Phellodendron* sp., *Phoebe* sp., *Quercus* sp., *Tilia* sp., etc. | N&S | NF | ≤40 | 41-60 | 61-80 | 81-120 | ≥121 |
|  | N&S | PF | ≤20 | 21-40 | 41-50 | 51-70 | ≥71 |

* State Forestry Administration (2004) Technical Regulation for National Forest Continuous Inventories. Beijing: State Forestry Administration. p.37.

† Mainland China is categorized by a demarcation line (Qinling Mountains-Huaihe River Line) into two regions: the North (N, the north of the Line, including cold- and warm-temperate zones) and the South (S, the south of the Line, including subtropical and tropical zones).

‡ Forests are categorized by stand origin into natural forest (NF) and planted forest (PF).

**Table S5** Statistics of stem biomass conversion factors (Mg m^-3^) by forest type and age class *

| Forest type (group) † | Young | Middle-aged | Premature | Mature | Overmature |
| --- | --- | --- | --- | --- | --- |
| All data | 0.521 ± 0.281 (437) | 0.492 ± 0.245 (434) | 0.517 ± 0.173 (95) | 0.487 ± 0.159 (100) | 0.484 ± 0.152 (33) |
| Forest type (group) |  |  |  |  |  |
| *Abies* and *Picea* | 0.642 ± 0.324 (11) | 0.502 ± 0.166 (10) | 0.539 ± 0.129 (6) | 0.339 ± 0.056 (6) | 0.407 ± 0.088 (4) |
| *Cunninghamia lanceolata* | 0.455 ± 0.232 (93) | 0.351 ± 0.069 (92) | 0.359 ± 0.063 (16) | 0.366 ± 0.069 (34) | 0.327 ± 0.021 (4) |
| *Cupressus* and *Fokienia* | 0.450 ± 0.101 (11) | 0.451 ± 0.110 (11) | NA | NA | NA |
| *Larix* | 0.553 ± 0.138 (37) | 0.481 ± 0.114 (42) | 0.480 ± 0.083 (3) | 0.524 (1) | 0.430 ± 0.051 (2) |
| *Pinus koraiensis* | 0.428 ± 0.082 (28) | 0.424 ± 0.089 (7) | NA | NA | NA |
| *P. massoniana* | 0.613 ± 0.274 (18) | 0.502 ± 0.143 (30) | 0.582 ± 0.115 (26) | 0.460 ± 0.118 (6) | NA |
| *P. tabuliformis* | 0.430 ± 0.104 (28) | 0.442 ± 0.089 (61) | 0.482 ± 0.163 (11) | 0.424 ± 0.014 (5) | NA |
| Other temperate pines and conifers | 0.645 ± 0.276 (13) | 0.544 ± 0.373 (25) | 0.692 ± 0.286 (3) | 0.543 (1) | 0.408 ± 0.160 (5) |
| Other subtropical pines and conifers | 0.473 ± 0.127 (31) | 0.404 ± 0.099 (31) | 0.476 ± 0.209 (5) | 0.451 ± 0.026 (2) | NA |
| *Betula* and *Populus* | 0.518 ± 0.289 (22) | 0.877 ± 0.647 (13) | 0.675 ± 0.072 (2) | 0.610 ± 0.117 (5) | 0.544 ± 0.142 (7) |
| Other deciduous broadleafs | 0.641 ± 0.201 (26) | 0.635 ± 0.200 (25) | 0.642 ± 0.276 (5) | 0.657 ± 0.122 (13) | 0.617 ± 0.158 (5) |
| *Acacia*, *Casuarina* and *Eucalyptus* | 0.567 ± 0.130 (45) | 0.684 ± 0.285 (40) | 0.548 ± 0.309 (3) | 0.635 ± 0.175 (6) | 0.421 (1) |
| Typical evergreen broadleafs | 0.601 ± 0.650 (27) | 0.545 ± 0.133 (21) | 0.512 ± 0.012 (3) | 0.557 ± 0.060 (3) | 0.715 ± 0.016 (2) |
| Other evergreen broadleafs | 0.879 ± 0.829 (9) | 0.731 ± 0.640 (9) | 0.414 ± 0.116 (5) | 0.574 ± 0.103 (4) | 0.462 ± 0.043 (3) |
| Mixed coniferous and broadleaved forest | 0.448 ± 0.167 (38) | 0.442 ± 0.128 (17) | 0.571 ± 0.235 (7) | 0.574 ± 0.182 (14) | NA |

* Data are means ± SD (standard deviation), and sample sizes are in parentheses. NA indicates no data available.

† Forest types (groups) are described in Table S1 in File S2.

**Table S6** Statistics of branch biomass conversion factors (Mg m^-3^) by forest type and age class *

| Forest type (group) † | Young | Middle-aged | Premature | Mature | Overmature |
| --- | --- | --- | --- | --- | --- |
| All data | 0.169 ± 0.129 (437) | 0.117 ± 0.088 (434) | 0.109 ± 0.088 (95) | 0.113 ± 0.109 (100) | 0.138 ± 0.105 (33) |
| Forest type (group) |  |  |  |  |  |
| *Abies* and *Picea* | 0.225 ± 0.166 (11) | 0.104 ± 0.049 (10) | 0.110 ± 0.037 (6) | 0.093 ± 0.019 (6) | 0.075 ± 0.041 (4) |
| *Cunninghamia lanceolata* | 0.146 ± 0.120 (93) | 0.053 ± 0.019 (92) | 0.045 ± 0.018 (16) | 0.038 ± 0.017 (34) | 0.023 ± 0.007 (4) |
| *Cupressus* and *Fokienia* | 0.113 ± 0.037 (11) | 0.109 ± 0.060 (11) | NA | NA | NA |
| *Larix* | 0.184 ± 0.123 (37) | 0.070 ± 0.029 (42) | 0.076 ± 0.027 (3) | 0.040 (1) | 0.033 ± 0.013 (2) |
| *Pinus koraiensis* | 0.155 ± 0.070 (28) | 0.078 ± 0.012 (7) | NA | NA | NA |
| *P. massoniana* | 0.298 ± 0.170 (18) | 0.108 ± 0.057 (30) | 0.078 ± 0.035 (26) | 0.083 ± 0.039 (6) | NA |
| *P. tabuliformis* | 0.198 ± 0.138 (28) | 0.160 ± 0.094 (61) | 0.132 ± 0.075 (11) | 0.108 ± 0.024 (5) | NA |
| Other temperate pines and conifers | 0.267 ± 0.137 (13) | 0.211 ± 0.102 (25) | 0.284 ± 0.095 (3) | 0.310 (1) | 0.188 ± 0.086 (5) |
| Other subtropical pines and conifers | 0.161 ± 0.072 (31) | 0.108 ± 0.077 (31) | 0.080 ± 0.053 (5) | 0.093 ± 0.073 (2) | NA |
| *Betula* and *Populus* | 0.179 ± 0.201 (22) | 0.224 ± 0.160 (13) | 0.199 ± 0.001 (2) | 0.309 ± 0.127 (5) | 0.177 ± 0.097 (7) |
| Other deciduous broadleafs | 0.183 ± 0.110 (26) | 0.172 ± 0.075 (25) | 0.191 ± 0.082 (5) | 0.261 ± 0.143 (13) | 0.220 ± 0.126 (5) |
| *Acacia*, *Casuarina* and *Eucalyptus* | 0.086 ± 0.069 (45) | 0.119 ± 0.100 (40) | 0.249 ± 0.313 (3) | 0.051 ± 0.026 (6) | 0.027 (1) |
| Typical evergreen broadleafs | 0.178 ± 0.128 (27) | 0.125 ± 0.074 (21) | 0.155 ± 0.041 (3) | 0.192 ± 0.096 (3) | 0.185 ± 0.158 (2) |
| Other evergreen broadleafs | 0.296 ± 0.234 (9) | 0.208 ± 0.104 (9) | 0.117 ± 0.035 (5) | 0.126 ± 0.091 (4) | 0.143 ± 0.054 (3) |
| Mixed coniferous and broadleaved forest | 0.157 ± 0.097 (38) | 0.118 ± 0.067 (17) | 0.124 ± 0.092 (7) | 0.114 ± 0.042 (14) | NA |

* Data are means ± SD (standard deviation), and sample sizes are in parentheses. NA indicates no data available.

† Forest types (groups) are described in Table S1 in File S2.

**Table S7** Statistics of foliage biomass conversion factors (Mg m^-3^) by forest type and age class *

| Forest type (group)† | Young | Middle-aged | Premature | Mature | Overmature |
| --- | --- | --- | --- | --- | --- |
| All data | 0.155 ± 0.198 (437) | 0.068 ± 0.065 (434) | 0.048 ± 0.051 (95) | 0.030 ± 0.025 (100) | 0.036 ± 0.027 (33) |
| Forest type (group) |  |  |  |  |  |
| *Abies* and *Picea* | 0.345 ± 0.457 (11) | 0.063 ± 0.043 (10) | 0.069 ± 0.013 (6) | 0.041 ± 0.007 (6) | 0.027 ± 0.021 (4) |
| *Cunninghamia lanceolata* | 0.281 ± 0.303 (93) | 0.054 ± 0.021 (92) | 0.034 ± 0.018 (16) | 0.023 ± 0.010 (34) | 0.009 ± 0.003 (4) |
| *Cupressus* and *Fokienia* | 0.142 ± 0.087 (11) | 0.080 ± 0.080 (11) | NA | NA | NA |
| *Larix* | 0.065 ± 0.042 (37) | 0.030 ± 0.012 (42) | 0.012 ± 0.002 (3) | 0.007 (1) | 0.007 ± 0.003 (2) |
| *Pinus koraiensis* | 0.119 ± 0.059 (28) | 0.032 ± 0.005 (7) | NA | NA | NA |
| *P. massoniana* | 0.160 ± 0.113 (18) | 0.054 ± 0.035 (30) | 0.027 ± 0.018 (26) | 0.018 ± 0.009 (6) | NA |
| *P. tabuliformis* | 0.190 ± 0.168 (28) | 0.105 ± 0.071 (61) | 0.070 ± 0.074 (11) | 0.036 ± 0.018 (5) | NA |
| Other temperate pines and conifers | 0.183 ± 0.109 (13) | 0.111 ± 0.069 (25) | 0.152 ± 0.156 (3) | 0.199 (1) | 0.070 ± 0.030 (5) |
| Other subtropical pines and conifers | 0.141 ± 0.101 (31) | 0.069 ± 0.046 (31) | 0.043 ± 0.026 (5) | 0.038 ± 0.021 (2) | NA |
| *Betula* and *Populus* | 0.089 ± 0.128 (22) | 0.121 ± 0.179 (13) | 0.149 ± 0.018 (2) | 0.063 ± 0.041 (5) | 0.060 ± 0.020 (7) |
| Other deciduous broadleafs | 0.073 ± 0.064 (26) | 0.043 ± 0.028 (25) | 0.043 ± 0.026 (5) | 0.027 ± 0.011 (13) | 0.022 ± 0.008 (5) |
| *Acacia*, *Casuarina* and *Eucalyptus* | 0.059 ± 0.075 (45) | 0.082 ± 0.100 (40) | 0.095 ± 0.107 (3) | 0.020 ± 0.012 (6) | 0.022 (1) |
| Typical evergreen broadleafs | 0.089 ± 0.083 (27) | 0.039 ± 0.022 (21) | 0.019 ± 0.006 (3) | 0.020 ± 0.010 (3) | 0.026 ± 0.013 (2) |
| Other evergreen broadleafs | 0.181 ± 0.274 (9) | 0.089 ± 0.083 (9) | 0.048 ± 0.032 (5) | 0.048 ± 0.033 (4) | 0.029 ± 0.003 (3) |
| Mixed coniferous and broadleaved forest | 0.131 ± 0.091 (38) | 0.056 ± 0.034 (17) | 0.046 ± 0.021 (7) | 0.029 ± 0.014 (14) | NA |

* Data are means ± SD (standard deviation), and sample sizes are in parentheses. NA indicates no data available.

† Forest types (groups) are described in Table S1 in File S2.

**Table S8** Statistics of aboveground biomass conversion factors (Mg m^-3^) by forest type and age class *

| Forest type (group) † | Young | Middle-aged | Premature | Mature | Overmature |
| --- | --- | --- | --- | --- | --- |
| All data | 0.846 ± 0.503 (437) | 0.679 ± 0.350 (434) | 0.676 ± 0.261 (95) | 0.633 ± 0.247 (100) | 0.660 ± 0.248 (33) |
| Forest type (group) |  |  |  |  |  |
| *Abies* and *Picea* | 1.212 ± 0.908 (11) | 0.672 ± 0.204 (10) | 0.719 ± 0.138 (6) | 0.479 ± 0.073 (6) | 0.515 ± 0.142 (4) |
| *Cunninghamia lanceolata* | 0.884 ± 0.586 (93) | 0.460 ± 0.094 (92) | 0.441 ± 0.080 (16) | 0.426 ± 0.089 (34) | 0.359 ± 0.019 (4) |
| *Cupressus* and *Fokienia* | 0.705 ± 0.133 (11) | 0.643 ± 0.238 (11) | NA | NA | NA |
| *Larix* | 0.802 ± 0.253 (37) | 0.581 ± 0.120 (42) | 0.568 ± 0.104 (3) | 0.572 (1) | 0.470 ± 0.067 (2) |
| *Pinus koraiensis* | 0.702 ± 0.191 (28) | 0.534 ± 0.095 (7) | NA | NA | NA |
| *P. massoniana* | 1.070 ± 0.475 (18) | 0.664 ± 0.214 (30) | 0.687 ± 0.142 (26) | 0.561 ± 0.143 (6) | NA |
| *P. tabuliformis* | 0.818 ± 0.365 (28) | 0.716 ± 0.244 (61) | 0.691 ± 0.226 (11) | 0.568 ± 0.016 (5) | NA |
| Other temperate pines and conifers | 1.100 ± 0.492 (13) | 0.871 ± 0.475 (25) | 1.129 ± 0.510 (3) | 1.052 (1) | 0.668 ± 0.250 (5) |
| Other subtropical pines and conifers | 0.778 ± 0.235 (31) | 0.582 ± 0.176 (31) | 0.600 ± 0.267 (5) | 0.582 ± 0.120 (2) | NA |
| *Betula* and *Populus* | 0.786 ± 0.608 (22) | 1.221 ± 0.914 (13) | 1.022 ± 0.090 (2) | 0.981 ± 0.167 (5) | 0.781 ± 0.238 (7) |
| Other deciduous broadleaved forests | 0.897 ± 0.321 (26) | 0.851 ± 0.265 (25) | 0.879 ± 0.333 (5) | 0.954 ± 0.216 (13) | 0.860 ± 0.266 (5) |
| *Acacia*, *Casuarina* and *Eucalyptus* | 0.714 ± 0.223 (45) | 0.888 ± 0.445 (40) | 0.895 ± 0.720 (3) | 0.711 ± 0.175 (6) | 0.492 (1) |
| Typical evergreen broadleafs | 0.868 ± 0.822 (27) | 0.709 ± 0.193 (21) | 0.686 ± 0.049 (3) | 0.769 ± 0.164 (3) | 0.927 ± 0.156 (2) |
| Other evergreen broadleafs | 1.356 ± 1.315 (9) | 1.027 ± 0.762 (9) | 0.579 ± 0.117 (5) | 0.748 ± 0.215 (4) | 0.635 ± 0.090 (3) |
| Mixed coniferous and broadleaved forest | 0.738 ± 0.285 (38) | 0.617 ± 0.207 (17) | 0.741 ± 0.308 (7) | 0.717 ± 0.214 (14) | NA |

* Data are means ± SD (standard deviation), and sample sizes are in parentheses. NA indicates no data available.

† Forest types (groups) are described in Table S1 in File S2.

**Table S9** Statistics of root biomass conversion factors (Mg m^-3^) by forest type and age class *

| Forest type (group) † | Young | Middle-aged | Premature | Mature | Overmature |
| --- | --- | --- | --- | --- | --- |
| All data | 0.214 ± 0.156 (313) | 0.151 ± 0.099 (312) | 0.1340 ± 0.090 (65) | 0.124 ± 0.062 (81) | 0.144 ± 0.076 (25) |
| Forest type (group) |  |  |  |  |  |
| *Abies* and *Picea* | 0.213 ± 0.144 (10) | 0.141 ± 0.036 (7) | 0.187 ± 0.160 (2) | 0.112 ± 0.047 (2) | 0.095 ± 0.051 (4) |
| *Cunninghamia lanceolata* | 0.260 ± 0.182 (65) | 0.105 ± 0.029 (80) | 0.093 ± 0.019 (12) | 0.081 ± 0.020 (33) | 0.071 ± 0.015 (4) |
| *Cupressus* and *Fokienia* | 0.158 ± 0.034 (8) | 0.128 ± 0.054 (10) | NA | NA | NA |
| *Larix* | 0.208 ± 0.069 (21) | 0.159 ± 0.054 (11) | 0.129 ± 0.020 (3) | 0.233 (1) | 0.165 ± 0.048 (2) |
| *Pinus koraiensis* | 0.168 ± 0.064 (26) | 0.112 ± 0.016 (7) | NA | NA | NA |
| *P. massoniana* | 0.209 ± 0.078 (16) | 0.126 ± 0.048 (25) | 0.109 ± 0.031 (17) | 0.086 ± 0.034 (5) | NA |
| *P. tabuliformis* | 0.149 ± 0.045 (27) | 0.159 ± 0.048 (33) | 0.124 ± 0.024 (9) | 0.129 ± 0.017 (5) | NA |
| Other temperate pines and conifers | 0.236 ± 0.098 (10) | 0.214 ± 0.206 (20) | 0.264 ± 0.163 (3) | 0.219 (1) | 0.224 ± 0.077 (5) |
| Other subtropical pines and conifers | 0.205 ± 0.097 (17) | 0.135 ± 0.082 (31) | 0.098 ± 0.033 (3) | 0.140 ± 0.002 (2) | NA |
| *Betula* and *Populus* | 0.132 ± 0.103 (17) | 0.251 ± 0.221 (9) | NA | 0.251 (1) | 0.236 (1) |
| Other deciduous broadleafs | 0.265 ± 0.127 (19) | 0.203 ± 0.074 (19) | 0.382 ± 0.031 (2) | 0.188 ± 0.074 (6) | 0.119 ± 0.017 (4) |
| *Acacia*, *Casuarina* and *Eucalyptus* | 0.174 ± 0.097 (32) | 0.184 ± 0.101 (18) | 0.220 ± 0.208 (3) | 0.146 ± 0.096 (5) | 0.031 (1) |
| Typical evergreen broadleafs | 0.342 ± 0.405 (18) | 0.185 ± 0.056 (20) | 0.120 ± 0.036 (2) | 0.115 ± 0.033 (2) | 0.250 (1) |
| Other evergreen broadleafs | 0.245 ± 0.099 (5) | 0.249 ± 0.230 (8) | 0.208 ± 0.199 (2) | 0.226 ± 0.046 (4) | 0.163 ± 0.046 (3) |
| Mixed coniferous and broadleaved forest | 0.208 ± 0.104 (22) | 0.144 ± 0.090 (14) | 0.152 ± 0.075 (7) | 0.152 ± 0.046 (14) | NA |

* Data are means ± SD (standard deviation), and sample sizes are in parentheses. NA indicates no data available.

† Forest types (groups) are described in Table S1 in File S2.

**Table S10** Statistics of whole biomass conversion factors (Mg m^-3^) by forest type and age class *

| Forest type (group) † | Young | Middle-aged | Premature | Mature | Overmature |
| --- | --- | --- | --- | --- | --- |
| All data | 1.049 ± 0.592 (313) | 0.807 ± 0.410 (312) | 0.816 ± 0.349 (65) | 0.703 ± 0.257 (81) | 0.730 ± 0.263 (25) |
| Forest type (group) |  |  |  |  |  |
| *Abies* and *Picea* | 1.476 ± 1.078 (10) | 0.869 ± 0.234 (7) | 0.791 ± 0.393 (2) | 0.588 ± 0.007 (2) | 0.610 ± 0.183 (4) |
| *Cunninghamia lanceolata* | 1.120 ± 0.687 (65) | 0.571 ± 0.119 (80) | 0.566 ± 0.078 (12) | 0.508 ± 0.100 (33) | 0.429 ± 0.032 (4) |
| *Cupressus* and *Fokienia* | 0.864 ± 0.181 (8) | 0.748 ± 0.284 (10) | NA | NA | NA |
| *Larix* | 1.026 ± 0.297 (21) | 0.823 ± 0.208 (11) | 0.697 ± 0.124 (3) | 0.805 (1) | 0.635 ± 0.115 (2) |
| *Pinus koraiensis* | 0.879 ± 0.246 (26) | 0.646 ± 0.109 (7) | NA | NA | NA |
| *P. massoniana* | 1.335 ± 0.541 (16) | 0.775 ± 0.168 (25) | 0.811 ± 0.146 (17) | 0.614 ± 0.152 (5) | NA |
| *P. tabuliformis* | 0.975 ± 0.375 (27) | 0.822 ± 0.196 (33) | 0.754 ± 0.198 (9) | 0.697 ± 0.024 (5) | NA |
| Other temperate pines and conifers | 1.122 ± 0.405 (10) | 1.107 ± 0.725 (20) | 1.393 ± 0.673 (3) | 1.271 (1) | 0.892 ± 0.325 (5) |
| Other subtropical pines and conifers | 0.915 ± 0.238 (17) | 0.717 ± 0.247 (31) | 0.740 ± 0.392 (3) | 0.721 ± 0.122 (2) | NA |
| *Betula* and *Populus* | 0.689 ± 0.209 (17) | 1.193 ± 1.051 (9) | NA | 1.482 (1) | 0.668 (1) |
| Other deciduous broadleafs | 1.139 ± 0.424 (19) | 1.031 ± 0.303 (19) | 1.442 ± 0.185 (2) | 0.962 ± 0.153 (6) | 0.872 ± 0.151 (4) |
| *Acacia*, *Casuarina* and *Eucalyptus* | 0.897 ± 0.327 (32) | 1.047 ± 0.458 (18) | 1.115 ± 0.928 (3) | 0.814 ± 0.249 (5) | 0.523 (1) |
| Typical evergreen broadleafs | 1.333 ± 1.386 (18) | 0.881 ± 0.225 (20) | 0.783 ± 0.005 (2) | 0.790 ± 0.007 (2) | 1.287 (1) |
| Other evergreen broadleafs | 1.267 ± 0.544 (5) | 1.324 ± 1.025(8) | 0.788 ± 0.359 (2) | 0.974 ± 0.259 (4) | 0.798 ± 0.135 (3) |
| Mixed coniferous and broadleaved forest | 1.034 ± 0.426 (22) | 0.769 ± 0.303 (14) | 0.893 ± 0.378 (7) | 0.869 ± 0.259 (14) | NA |

* Data are means ± SD (standard deviation), and sample sizes are in parentheses. NA indicates no data available.

† Forest types (groups) are described in Table S1 in File S2.
